# The Clinical Profile of Patients With Acute Decompensated Heart Failure Presenting to the Emergency Department at a Tertiary Care Hospital in India

**DOI:** 10.7759/cureus.67773

**Published:** 2024-08-25

**Authors:** Varsha Shinde, Dhruvkumar Thakkar, Sharmila J Mavudelli

**Affiliations:** 1 Emergency Medicine, Dr. D. Y. Patil Medical College, Hospital, and Research Centre, Dr. D. Y. Patil Vidyapeeth (Deemed to be University), Pune, IND

**Keywords:** dyspnea, heart failure, epidemiological profile, clinical profile, emergency department, acute decompensated heart failure (adhf)

## Abstract

Background

Acute decompensated heart failure (ADHF) poses a significant burden on healthcare systems globally, including in India, due to its high morbidity and mortality rates. This study aimed to evaluate the clinical characteristics of patients presenting with ADHF to the emergency department (ED) of a tertiary care hospital in India.

Methodology

This observational study was conducted at Dr. D. Y. Patil Medical College, Hospital, and Research Centre in Pune, India. Ninety patients aged 12 years and older who presented with signs and symptoms of heart failure (HF) to the ED between January 2023 and March 2024 were enrolled as participants. Ethical approval was obtained. Written consent was obtained from all participants. Clinical diagnoses were based on patient history, physical examination, chest radiograph, point-of-care ultrasound (POCUS), electrocardiography (ECG), echocardiography, and radiological and laboratory findings. Data were analyzed using IBM SPSS Statistics version 29.0.2.0 (Armonk, NY: IBM Corp.) and represented as mean±SD, frequency (n), and percentage.

Results

The study involved 90 participants with a mean age of 61.1±16.3 years. The cohort comprised 51 males (56.7%) and females 39 (43.3%). Dyspnea was the most common clinical presentation in all participants, followed by swelling of feet in 58 (64.4%) cases. The mean systolic blood pressure noted was 142.1±42.8 mmHg. Hypertension was the most frequently identified risk factor, present in 52 (57.8%) cases. The most common precipitating factor identified was anemia in 39 (43.3%) cases. Point-of-care ultrasonography (pulmonary) revealed significant B-lines (≥2 of the eight thoracic zones with ≥3 B-lines or B-line count in all eight zones ≥10) in 85 (94.4%) cases. B-type natriuretic peptide (BNP) was elevated in all participants. The mean hemoglobin levels in males and females were 13.2±2.6 g/dL and 10.6±2.8 g/dL, respectively. The mean serum sodium level was 132.4±6.2 mEq/dL. Serum sodium level below 135.0 mEq/L (hyponatremia) was found in 53 (58.9%) cases. The mean serum creatinine level was 1.7±1.4 mg/dL. Diuretics were the most common treatment modality used in the ED. More than half of the patients (72.2%) were transferred to the intensive care unit; the mortality rate in the ED was 2.2%.

Conclusion

This study provides comprehensive insights into the characteristics, management, and outcomes of ADHF patients presenting to the ED of a tertiary care hospital in India. The findings highlight the challenges and complexities in managing ADHF in this population and underscore the need for tailored therapeutic approaches to improve patient outcomes and reduce healthcare utilization.

## Introduction

Heart failure (HF) is characterized by a dysfunction in cardiac performance, leading to the heart’s inability to pump blood effectively to meet the body’s metabolic demands [1]. Acute decompensated heart failure (ADHF) is defined as the abrupt onset of HF symptoms that require urgent medical attention, often resulting in unplanned visits to medical facilities, emergency rooms, or hospital admissions [2]. These symptoms typically result from severe pulmonary congestion caused by increased left ventricular filling pressures, with or without reduced cardiac output [2].

HF is a growing global health concern, affecting over 20 million people worldwide, including more than five million in the United States [3]. The incidence of HF increases exponentially with age [3]. In India, the prevalence of HF is estimated to range between 1.3 million and 4.6 million cases, with an annual incidence of 491,600-1.8 million new cases. Population dynamics, shifts in epidemiology, and healthcare transitions are expected to significantly influence the future prevalence of HF in India. Risk factors such as hypertension, coronary artery disease, diabetes, obesity, and rheumatic heart disease contribute to the development of HF. The rising prevalence of these risk factors, coupled with increased life expectancy and improved management of chronic conditions, has led to the growing incidence of HF [4,5].

Most hospital admissions for ADHF involve the exacerbation of pre-existing chronic heart failure, while approximately 15-20% of admissions are due to newly diagnosed HF [6]. Patients with newly diagnosed HF often present with pulmonary edema or cardiogenic shock; those with worsening chronic heart failure typically show signs of fluid overload and congestion, including weight gain, exertional dyspnea, or difficulty breathing when lying down. These symptoms typically develop over days or weeks before seeking medical attention [6].

In developing countries, HF significantly contributes to morbidity and mortality. Unlike in the Western world, the epidemiology of HF in developing nations, such as India, remains an active area of research, with comprehensive studies assessing its burden still lacking. However, the impact is expected to be significant due to India's large share of the global population (16%), its burden of a quarter of the world's coronary heart disease (CHD), 120 million individuals with hypertension, and a significant number of people affected by rheumatic heart disease (RHD) [4,5].

This study aimed to investigate the clinical characteristics of patients with ADHF, covering demographics (age and sex), etiology (risk factors and precipitating factors), initial presentations (signs and symptoms), laboratory and radiological results, treatment modalities, and early outcomes in the emergency department (ED).

## Materials and methods

Study design and setting

This observational study was conducted at Dr. D. Y. Patil Medical College, Hospital, and Research Centre in Pune, India. Ninety patients over 12 years of age presenting with signs and symptoms of HF to the ED from January 2023 to March 2024 were included. Each participant underwent a thorough clinical assessment and investigation. The study was approved by the Research and Recognition Committee of Dr. D. Y. Patil Medical College, Hospital, and Research Centre, Pune, India (#IESC/PGS/2022/179). Consent forms, written in the participants’ native languages, were provided to ensure that they understood the study's objectives, procedures, and possible risks.

Patients over 12 years of age presenting with HF signs and symptoms were included in the study. Exclusion criteria were patients under 12 years of age, those unwilling to be hospitalized, and those who did not consent to participate.

Sample size

The sample size of 90 was calculated using WinPepi software version 11.38 (Brixton Health, United Kingdom: J. H. Abramson). The estimated sample size was based on the prevalence (68.9%) of ischemic etiology in ADHF as observed by Onteddu et al., with a 95% confidence interval and an acceptable difference of 10% [7].

Data and sample collection

Samples were chosen using predetermined inclusion and exclusion criteria. Clinical profiles were examined through history, physical examination, chest radiograph, electrocardiography (ECG), echocardiography, and point-of-care ultrasound (POCUS). Point-of-care pulmonary ultrasound was performed by an emergency physician on all patients to detect significant B-lines (≥2 of the eight thoracic zones with ≥3 B-lines or B-line count in all eight zones ≥10) [8]. Laboratory investigations included B-type natriuretic peptide (BNP), complete hemogram, liver function test (LFT), renal function test (RFT), serum electrolytes (SE), blood sugar level (BSL), HIV, hepatitis-B surface antigen (HBsAg), anti-hepatitis C virus (anti-HCV) antibody, creatinine kinase-MB (CK-MB), and troponin-I. Statistical methods were used to conduct a thorough examination of all these parameters.

Statistical analysis

Statistical analysis was performed using IBM SPSS Statistics version 29.0.2.0 (Armonk, NY: IBM Corp.) software. Continuous variables were expressed as mean±SD, while categorical variables were expressed as frequencies (n) and percentages. Statistical significance and hypothesis testing were not considered. Data tracking used Microsoft Excel (Redmond, WA: Microsoft Corp.).

## Results

The study included 90 patients with a mean age of 61.1 years, comprising 56.7% males and 43.3% females. Mean vital signs were as follows: respiratory rate 30.9 breaths/min, oxygen saturation (SpO_2_) 82.6%, systolic blood pressure 142.1 mmHg, and pulse rate 106.8 bpm. All participants had dyspnea. Additionally, 64.4% had swelling of feet, 36.7% chest pain, 77.8% elevated jugular venous pressure (JVP), and 64.4% edema. Detailed demographics, vital signs, and clinical presentations are described in Table 1.

**Table 1:** Demographics, vital signs, and clinical presentations of patients with ADHF. *Continuous variables are presented as mean±SD and categorical variables are presented as frequency (n) and percentage. JVP: jugular venous pressure; N: total number of patients; ADHF: acute decompensated heart failure

Variables		N=90
Patient characteristic	Age (year)* (mean±SD)	61.1±16.2
	Gender	
	Male (year)* (mean±​​​​​​SD)	60.1±17.7
	Female (year)* (mean±​​​​​​SD)	62.4±14.3
	Number of patients	
	Male, n (%)	51 (56.7)
	Female, n (%)	39 (43.3)
Vital signs	Respiratory rate (breaths/min)* (mean±​​​​​​SD)	30.9±8.89
	SPO_2_ (%)* (mean±​​​​​​SD)	82.6±12.7
	Systolic (mmHg)* (mean±​​​​​​SD)	142.1±42.8
	Diastolic (mmHg)* (mean±​​​​​​SD)	84.2±19.9
	Pulse (bpm)* (mean±​​​​​​SD)	106.8±22.8
Clinical presentations	Dyspnea, n (%)	90 (100.0)
	Swelling of feet, n (%)	58 (64.4)
	Chest pain, n (%)	33 (36.7)
	Cough, n (%)	33 (36.7)
	Fatigue, n (%)	14 (15.6)
	Fever, n (%)	12 (13.3)
	Palpitation, n (%)	7 (7.8)
	Abdominal discomfort, n (%)	3 (3.3)
	Syncope, n (%)	2 (2.2)
	Elevated JVP, n (%)	70 (77.8)
	Edema, n (%)	58 (64.4)

The mean left ventricular ejection fraction (LVEF) was 44.6±14.8%. Of the 90 patients, 85 (94.4%) had significant B-lines [8]. The New York Heart Failure Association’s (NYHA) functional class distribution was 10 (11.1%) in class II, 36 (40.0%) in class III, and 44 (48.9%) in class IV. Patients were classified by LVEF as follows: 42 (46.7%) with LVEF >50.0% (heart failure with preserved ejection fraction {HFpEF}), 11 (12.2%) with LVEF 40.0-49.0% (heart failure with mildly reduced ejection fraction {HFmrEF}), and 37 (41.1%) with LVEF <40.0% (heart failure with reduced ejection fraction {HFrEF}) (Table 2).

**Table 2:** Characteristics and classifications (according to NYHA and LVEF) of patients with ADHF. *Continuous variables are presented as mean±SD and categorical variables are presented as frequency (n) and percentage. LVEF: left ventricular ejection fraction; NYHA: New York Heart Failure Association; HFpEF: heart failure with preserved ejection fraction; HFmrEF: heart failure with mildly reduced ejection fraction; HFrEF: heart failure with reduced ejection fraction; ADHF: acute decompensated heart failure; N: total number of patients

Characteristics	N=90
LVEF (%)* (mean±SD)	44.6±14.8
Significant B-lines, n (%)	85 (94.4%)
NYHA classification, n (%)	
Class II	10 (11.1)
Class III	36 (40.0)
Class IV	44 (48.9)
Based on LVEF, n (%)	
HFpEF (LVEF >50.0%)	42 (46.7)
HFmrEF (LVEF 40.0-49.0%)	11 (12.2)
HFrEF (LVEF <40.0%)	37 (41.1)

The most common risk factor associated with ADHF identified was hypertension (57.8%), followed by diabetes (38.9%), and ischemic heart disease (30%). Non-rheumatic valvular heart disease was present in 25.6%. Other associated risk factors are mentioned in Table 3. Among the various precipitating factors, anemia was the most common, observed in 43.3% of cases (Table 4).

**Table 3:** Risk factors in patients with ADHF. Categorical variables are presented as frequency (n) and percentage. PCI: Percutaneous intervention; CABG: Coronary artery bypass grafting; N: total number of patients; ADHF: acute decompensated heart failure

Risk factors, N=90	n (%)
Diabetes	35 (38.9)
Hypertension	52 (57.8)
Ischemic heart disease	27 (30.0)
PCI/CABG	15 (16.7)
Atrial fibrillation	7 (7.8)
Cerebral vascular accident	4 (4.4)
Chronic kidney disease	4 (4.4)
Chronic liver disease	1 (1.1)
Chronic obstructive pulmonary disease	4 (4.4)
Bronchial asthma	3 (3.3)
Interstitial lung disease	2 (2.2)
Congenital heart disease	1 (1.1)
Dilated cardiomyopathy	9 (10.0)
Rheumatic valvular heart disease	7 (7.8)
Non-rheumatic valvular heart disease	23 (25.6)
Smoking	17 (18.9)
Alcohol	18 (20.0)

**Table 4:** Precipitating factors in patients with ADHF. Categorical variables are presented as frequency (n) and percentage. STEMI: ST-elevation myocardial infarction; NSTEMI: non-ST-elevation myocardial infarction; LBBB: left bundle branch block; OAD: obstructive airway disease; COPD: chronic obstructive pulmonary disease; BA: bronchial asthma; N: total number of patients; ADHF: acute decompensated heart failure

Precipitating factors, N=90	n (%)
New cardiac events (STEMI/NSTEMI/new-onset LBBB)	31 (34.4)
Anemia	39 (43.3)
Infection	26 (28.9)
Diabetic ketoacidosis	2 (2.2)
Hypothyroidism	1 (1.1)
Drug non-compliance	30 (33.3)
Worsening renal function	15 (16.7)
Uncontrolled hypertension	25 (27.8)
Exacerbation of OAD (BA/COPD)	8 (8.9)
Prosthetic valvular disease	3 (3.3)
Atrial fibrillation	7 (7.8)

Table 5 presents the laboratory findings of patients with ADHF. All patients had positive BNP (>100.0 pg/mL). In this study, 53 participants (58.9%) had hyponatremia (serum sodium below 135.0 mEq/L) with a mean serum sodium of 132.4±6.2 mEq/L. The mean serum creatinine was 1.7±1.4 mg/dL. The mean hemoglobin levels were 13.2±2.56 g/dL in males and 10.6±2.8 g/dL in females. Various treatment modalities were used in the ED (Table 6). The most common treatment administered to patients was diuretic (95.6%) followed by oxygen supplementation (86.7%).

**Table 5:** Laboratory tests performed in patients with ADHF. *Continuous variables are presented as mean±SD and categorical variables are presented as frequency (n) and percentage. BNP: B-type natriuretic peptide; N: total number of patients; ADHF: acute decompensated heart failure

Laboratory tests	N=90	Reference range
BNP >100 pg/mL, n (%)	90.0 (100.0) pg/mL	<100.0 pg/mL
Sodium (mEq/L)* (mean±SD)	132.4±6.2 mEq/L	135.0-145.0 mEq/L
Potassium (mEq/L)* (mean±SD)	4.4±0.8 mEq/L	3.5-5.1 mEq/L
Chloride (mEq/L)* (mean±SD)	101.3±6.4 mEq/L	96.0-106.0 mEq/L
Calcium (mg/dL)* (mean±SD)	8.5±0.56 mg/dL	8.5-10.5 mg/dL
Phosphate (mg/dL)* (mean±SD)	4.7±0.8 mg/dL	2.5-4.5 mg/dL
Magnesium (mg/dL)* (mean±SD)	2.2±0.5 mg/dL	1.7-2.2 mg/dL
Hemoglobin (g/dL)* (mean±SD)	Male 13.2±2.6 g/dL	14.0-18.0 g/dL
	Female 10.6±2.8 g/dL	12.0-16.0 g/dL
Serum Creatinine (mg/dL)*	1.7±1.4 mg/dL	0.7-1.3 mg/dL

**Table 6:** Treatment modalities used in patients with ADHF in the ED. Categorical variables are presented as frequency (n) and percentage. NTG: nitroglycerin; ACE: angiotensin-converting enzyme; ARB: angiotensin receptor blocker; LMWH: low molecular weight heparin; UFH: unfractionated heparin; NIV: non-invasive ventilation; N: total number of patients; ADHF: acute decompensated heart failure

Treatment modalities, N=90	n (%)
NTG	25 (27.8)
Diuretic	86 (95.6)
Oxygen supplement	78 (86.7)
IV fluids	6 (6.7)
Beta-blocker	17 (18.9)
Calcium channel blocker	7 (7.8)
Digoxin	2 (2.2)
ACE inhibitor	3 (3.3)
ARB	20 (22.2)
LMWH or UFH	39 (43.3)
Thrombolytics	9 (10.0)
Statin	63 (70.0)
Anti-platelets	62 (68.9)
Noradrenaline	7 (7.8)
Dobutamine	6 (6.7)
Dopamine	2 (2.2)
Amiodarone	5 (5.6)
Adenosine	1 (1.1)
Invasive ventilation	5 (5.6)
NIV	40 (44.4)
Antibiotics	51 (56.7)
Nebulization	12 (13.3)

Of the 90 patients, 65 (72.2%) were transferred to the intensive care unit (ICU), while 23 (25.6%) were moved to a ward. The mortality rate in the ED was 2.2%.

## Discussion

This study offers descriptive data on individuals with ADHF in a tertiary care hospital in India. It offers preliminary insights into the epidemiology, clinical presentation, risk factors, precipitating factors, treatment modalities, and outcomes of ADHF patients in the ED.

When compared with various Western studies such as the Euro Heart Failure Survey-1 (EHFS-1), Euro Heart Failure Survey-2 (EHFS-2), United States Acute Decompensated Heart Failure National Registry (US ADHERE), Organized Program to Initiate Life-Saving Treatment in Hospitalized Patients With Heart Failure (OPTIMIZE-HF), and Acute Decompensated Heart Failure Study (AFAR) represented in Table 7, we observed that Indians were younger and experienced the effect of cardiovascular disease earlier in life [9-15]. Males accounted for 56.7% of the patients in the present study, whereas the percentages in US ADHERE, OPTIMIZE-HF, EFHS-1, EFHS-2, and AFAR were 48%, 48%, 53%, 61%, and 63%, respectively [9-15] (Table 7).

**Table 7:** Comparison of age and sex distribution among various studies. *Continuous variables are presented as mean±SD and categorical variables are presented as frequency (n) and percentage. EHFS-1: Euro Heart Failure Survey-1; EHFS-2: Euro Heart Failure Survey-2; US ADHERE: United States Acute Decompensated Heart Failure National Registry; OPTIMIZE-HF: Organized Program to Initiate Life-Saving Treatment in Hospitalized Patients With Heart Failure; AFAR: Acute Decompensated Heart Failure Study; N: total number of patients

Study	Age (year)*, mean±SD	Male, n (%)
The present study (N=90)	61.1±16.2	51 (56.7)
US ADHERE (N=1,05,388) [12]	72.4±14.0	50,586 (48.0)
OPTIMIZE-HF (N=48,612) [13]	73.1±14.2	23,334 (48.0)
EHFS-1 (N=11.327) [9]	71.0±12.2	6,003 (53.0)
EHFS-2 (N=3,580) [10,11]	69.9±12.5	2,184 (61.0)
AFAR (N=90) [15]	53.5±17.7	57 (63.0)

In this study, the most common clinical presentation found was dyspnea, observed in 100% of the patients. Similar findings were noted in other studies, such as AFAR (79.7%) and Thai ADHERE (96.7%) [15,16]. The clinical presentations across various studies, including the present study, AFAR, and Thai ADHERE, are detailed in Table 8.

**Table 8:** Comparison of clinical presentations among various studies. Categorical variables are presented as frequency (n) and percentage. AFAR: Acute Decompensated Heart Failure Study; Thai ADHERE: Thailand Acute Decompensated Heart Failure National Registry; N: total number of patients

Clinical presentation, n (%)	Our study, N=90	AFAR, N=90 [15]	Thai ADHERE, N=2,041 [16]
Dyspnea	90 (100.0)	71 (79.7)	1,973 (96.7)
Fatigue	14 (15.6)	53 (59.4)	735 (36.0)
Chest pain	33 (36.7)	29 (32.9)	-
Peripheral edema	58 (64.4)	21 (23.4)	1,215 (59.5)

Undifferentiated dyspnea is a common presentation in the ED. Pulmonary ultrasonography plays a crucial role in differentiating the causes of dyspnea, such as acute ADHF, pneumonia, and obstructive airway diseases (OAD), enabling the initiation of early treatment. The presence of significant B-lines (in two of the eight thoracic zones with ≥3 B-lines or B-line count in all eight zones ≥10) is suggestive of pulmonary congestion [8]. Lichtenstein and Mezière found that multiple B-lines dispersed over both anterolateral lungs had a 100% sensitivity and 92% specificity for diagnosing pulmonary edema [8]. In the second study, the “BLUE protocol” (bedside lung ultrasound in emergency) was assessed, with ICU staff comparing lung ultrasonography results to the final diagnosis. Diffuse multiple B-lines were shown to be 97% sensitive and 95% specific for cardiogenic pulmonary edema [17]. In the present study, 85 patients (94.4%) exhibited significant B-lines on pulmonary ultrasonography.

We also compared common underlying risk factors across various studies, including Thai ADHERE, US ADHERE, EHFS-1, EHFS-2, OPTIMIZE-HF, and the present study [9-14,16] (Table 9). Hypertension was found to be the most common associated risk factor in almost all studies. Other underlying risk factors identified in the present study included congenital heart disease (1.1%), dilated cardiomyopathy (10%), rheumatic valvular heart disease (7.8%), non-rheumatic valvular disease (25.6%), smoking (18.9%), and alcohol use (20%).

**Table 9:** Comparison of risk factors among various studies. Categorical variables are presented as frequency (n) and percentage. EHFS-1: Euro Heart Failure Survey-1; EHFS-2: Euro Heart Failure Survey-2; US ADHERE: United States Acute Decompensated Heart Failure National Registry; OPTIMIZE-HF: Organized Program to Initiate Life-Saving Treatment in Hospitalized Patients With Heart Failure; Thai ADHERE: Thailand Acute Decompensated Heart Failure National Registry; TIA: transient ischemic attack; COPD: chronic obstructive pulmonary disease; BA: bronchial asthma

Risk factors, n (%)	Our study, N=90	Thai ADHERE, N=2,041 [16]	US ADHERE, N=1,05,388 [12]	EHFS-1, N=11,327 [9]	EFHS-2, N=3,580 [10,11]	OPTIMIZE-HF, N=48,612 [13]
Diabetes	35 (38.9)	966 (47.3)	46,371 (44.0)	3,058 (27.0)	1,181 (33.0)	20,417 (42.0)
Hypertension	52 (57.8)	1,322 (64.8)	76,933 (73.0)	6,003 (53.0)	2,183 (61.0)	34,514 (71.0)
Ischemic heart disease	27 (40.0)	951 (46.6)	60,017 (57.0)	7,702 (68.0)	1,933 (54.0)	24,306 (50.0)
Atrial fibrillation	7 (7.8)	491 (24.1)	32,670 (31.0)	4,757 (42.0)	1,396 (39.0)	15,069 (31.0)
Stroke/TIA	4 (4.4)	246 (12.1)	17,916 (17.0)	2,152 (19.0)	465 (13.0)	7,778 (16.0)
Chronic kidney disease	4 (4.4)	396 (19.4)	31,616 (30.0)	1,925 (17.0)	608 (17.0)	9,722 (20.0)
COPD/BA	7 (7.8)	161 (7.9)	58,145 (31.0)	3,624 (30.2)	680 (19.0)	16,528 (34.0)

In this study, anemia was identified as the most common precipitating factor, consistent with findings from the study by Kaler et al. [18]. The Thai ADHERE study identified a new cardiac event as the most common precipitating factor [16]. Table 10 compares the precipitating factors found in the present study with those reported by Kaler et al. and the Thai ADHERE study [16,18].

**Table 10:** Comparison of precipitating factors among various studies. Categorical variables are presented as frequency (n) and percentage. Thai ADHERE: Thailand Acute Decompensated Heart Failure National Registry; STEMI: ST-elevation myocardial infarction: NSTEMI: non-ST-elevation myocardial infarction; LBBB: left bundle branch block; OAD: obstructive airway disease; N: total number of patients

Precipitating factor, n (%)	Our study, N=90	Kaler et al., N=150 [18]	Thai ADHERE, N=041 [16]
New cardiac events (STEMI/NSTEMI/new-onset LBBB)	31 (34.4)	71 (47.3)	651 (31.9)
Anemia	39 (43.3)	96 (63.8)	-
Infection	26 (28.9)	53 (35.3)	443 (21.7)
Atrial fibrillation	7 (7.8)	30 (20.0)	-
Drug non-compliance	30 (33.3)	28 (18.7)	88 (4.3)
Hypothyroidism	1 (1.1)	6 (4.0)	-
Worsening renal function	15 (16.7)	-	484 (23.7)
Uncontrolled hypertension	25 (27.8)	17 (11.3)	-
Exacerbation of OAD	8 (8.9)	-	-
Inadequate diuresis	-	-	151 (7.4)
New medication use	-	-	35 (1.7)
Prosthetic valvular disease	3 (3.3)	-	-

We classified the patients with ADHF according to the NYHA functional classification as follows: class II (11.1%), class III (40.0%), and class IV (48.9%). The Thai ADHERE study reported a distribution of NYHA classes among ADHF patients, with 14.7% classified as class II, 16.2% as class III, and the majority (69.1%) as class IV, indicating a high proportion of patients with severe symptoms and advanced heart failure [16].

Patients were also classified based on left ventricular ejection fraction into three classes as follows: HFpEF (LVEF: >50.0%) in 46.7%, HFmrEF (LVEF: 41.0-49.0%) in 12.2%, and HFrEF (LVEF: <40.0%) in 41.1% of patients. Chang et al. investigated that, of the hospitalizations for ADHF, 39.0% had preserved EF (HFpEF), 11.0% had mildly reduced EF (HFmrEF), and 50.0% had a reduced ejection fraction (HFrEF) [19]. The mean LVEF in the present study was 44.6±14.8%. The prevalence of reduced left ventricular ejection fraction (LVEF <40.0%) in patients with ADHF was consistently observed across various studies, with rates of 43.6% in the Thai ADHERE study, 46.0% in the US ADHERE study, 45.0% in the EFHS-1 study, and 41.1% in the present study. All four studies suggested that over half of heart failure patients had an LVEF of >40.0% [9,12,16].

Hemoglobin measurement is crucial in acute heart failure due to its prognostic significance. Low hemoglobin levels are linked to poor outcomes, including increased morbidity and mortality in HF patients. In our study, the mean hemoglobin levels in males and females were 13.2±2.6 g/dL and 10.6±2.8 g/dL, respectively. Additionally, 17 males and 22 females had hemoglobin levels <14.0 g/dL and <12.0 g/dL, respectively. One study found that patients with HFpEF and anemia (hemoglobin below 12.0 g/dL) had a 2.1-fold higher mortality risk and a 3.5-day increase in hospital stay compared to those without anemia. Patients with HFrEF and anemia (hemoglobin level below 12.0 g/dL) had a 1.4-fold higher mortality risk and a 1.8-day increase in hospital stay compared to those without anemia [20].

ADHF patients are at risk of hyponatremia (<135.0 mEq/L), which can worsen the heart failure symptoms. Also, it has prognostic significance, as hyponatremia is associated with poor outcomes, increased morbidity, and mortality. The mean sodium level calculated was 132.4±6.2 mEq/L. Out of 90 patients, 53 patients had hyponatremia (serum sodium <135.0 mEq/L). In Thai ADHERE study, the mean sodium level found was 136.8 mEq/L with 9.9% of patients having serum sodium levels <130.0 mEq/L [16]. In the OPTIMIZE-HF study, 20% of patients had hyponatremia (<135.0 mEq/L) [13,14]. Hyponatremia in this condition may arise from two opposing processes as follows: either due to volume overload leading to dilutional hyponatremia or from hypovolemic hyponatremia resulting from excessive diuretic use.

Renal function assessment is crucial in ADHF patients, as ADHF can lead to kidney damage or worsen existing kidney disease or vice versa. It is also useful for diuretic therapy guidance and fluid management. Renal function is a strong predictor of outcomes in ADHF patients. Measuring renal function helps to identify high-risk patients. In the AFAR study, OPTIMIZE-HF, and the present study, the mean serum creatinine levels were 1.5±0.8 mg/dL, 1.8±1.8 mg/dL, and 1.7±1.4 mg/dL, respectively [13-15]. In the present study, 15.0 patients (16.7%) experienced worsening renal function (serum creatinine >2.0 mg/dL), while 23.7% of patients in the Thai ADHERE study showed similar findings [16].

Various treatment modalities are available for managing acute decompensated heart failure (ADHF). In the present study, the most administered treatment was diuretic (95.6%), consistent with the findings from the Thai ADHERE study [16]. Non-invasive ventilation emerged as a crucial treatment modality in the ED due to its beneficial effects, such as reduction in respiratory distress, correction of metabolic imbalances, decreased need for intubation, and improved fluid distribution compared to standard therapy alone. However, its impact on mortality remains uncertain. In the present study, nearly half of the patients received non-invasive ventilation support.

We categorized outcomes for ADHF patients in the emergency department as follows: intensive care unit admission, ward admission, or death. We found that 65 patients (72.2%) were transferred to the intensive care unit (ICU), 23 patients (25.6%) were admitted to the ward for further management, and the mortality rate in the emergency department was 2 (2.2%).

Limitations

This study's single-hospital setting may introduce selection bias due to unique patient demographics and clinical practices, limiting the findings' applicability to other populations or settings. Additionally, the sample size may be insufficient to identify less common clinical presentations, risk factors, and precipitating factors, potentially restricting the scope of our conclusions. As the research was conducted in the ED, long-term outcomes could not be assessed. Furthermore, the study's descriptive nature and lack of a control group prevented the use of statistical analyses, such as calculating p-values to compare groups and assess the statistical significance of the results.

## Conclusions

This study provides insights into the clinical profile of patients with ADHF presenting to the ED. Our findings suggest that Indian patients admitted for HF tend to be younger and present with more severe illness compared to patients in Western countries. Male patients were more prevalent than female patients, and heart failure with preserved ejection fraction (HFpEF) was highly prevalent. Anemia was identified as the predominant underlying precipitating factor for HF, followed by a new cardiac event (ST-elevation myocardial infarction {STEMI}/non-ST-elevation myocardial infarction {NSTEMI}/new-onset LBBB), while hypertension emerged as the most prevalent underlying risk factor. Dyspnea was found to be the most common clinical presentation, followed by peripheral edema, and more than half of the patients had hyponatremia. These findings offer valuable insights for clinicians managing patients with ADHF, aiming to improve patient outcomes and reduce mortality in the ED.
